# Predictors of Change in CD4 Lymphocyte Count and Weight among HIV Infected Patients on Anti-Retroviral Treatment in Ethiopia: A Retrospective Longitudinal Study

**DOI:** 10.1371/journal.pone.0058595

**Published:** 2013-04-03

**Authors:** Ayalu A. Reda, Sibhatu Biadgilign, Amare Deribew, Betemariam Gebre, Kebede Deribe

**Affiliations:** 1 Population Studies and Training Center (PSTC), Brown University, Providence, Rhode Island, United States of America; 2 Department of Epidemiology and Biostatistics, College of Public Health and Medical Science, Jimma University, Jimma, Ethiopia; 3 Medical Emergency Relief International (Merlin), Country Office, Addis Ababa, Ethiopia; 4 Brighton and Sussex Medical School, Falmer, Brighton, United Kingdom; Istituto Superiore di Sanità, Italy

## Abstract

**Background:**

Antiretroviral treatment (ART) has been introduced in Ethiopia a decade ago and continues to be scaled up. However, there is dearth of literature on the impact of ART on changes in CD4 lymphocyte count and weight among patients on treatment.

**Objective:**

To determine the predictors of change in CD4 lymphocyte count and weight among HIV/AIDS infected patients taking antiretroviral treatment in eastern Ethiopia.

**Methods:**

A retrospective cohort study was conducted among HIV/AIDS patients taking ART from 2005 to 2010. A sample of 1540 HIV infected adult patients who started antiretroviral therapy in hospitals located in eastern Ethiopia were included in the study. The primary outcomes of interest were changes in CD4 count and weight. Descriptive statistics and multivariable regression analyses were performed to examine the outcomes among the cohort.

**Results:**

Both the median CD4 lymphocyte counts and weight showed improvements in the follow up periods. The multivariate analysis shows that the duration of ART was an important predictor of improvements in CD4 lymphocyte count (beta 7.91; 95% CI 7.48–8.34; p 0.000) and weight (beta 0.15; 95% CI 0.13–0.18; p 0.000). Advanced WHO clinical stage, lower baseline CD4 cell count, and baseline hemoglobin levels were factors associated with decline in weight. Actively working patients had higher CD4 lymphocyte count and weight compared to those that were ambulatory (p<0.05).

**Conclusion:**

We detected a substantial increment in weight and CD4 lymphocyte count among the patients who were taking ART in eastern Ethiopia. Patients who are of older age, with low initial CD4 lymphocyte count, late stage of the WHO clinical stages and lower hemoglobin level may need special attention. The reasons for the improved findings on CD4 count and weight throughout the five years of follow up merit further investigation.

## Introduction

HIV/AIDS continues to be a global challenge. Sub-Sahara Africa is the most affected part of the world with an estimated 22.9 million people living with HIV and 1.2 million deaths from AIDS among children and adults in 2010 [1]. Immunological suppression and disturbance caused by HIV infection is responsible for the decline in CD4 cell counts and is predictive of both morbidity and mortality from AIDS [2], [3].

The introduction of antiretroviral treatment has altered the course of the HIV/AIDS epidemic [4], [5]. Antiretroviral drugs control the HIV infection by reducing viral replication which in most cases is measured by increases in CD4 lymphocyte count [3]. This has led to drastic reductions in morbidity and mortality from AIDS, and improvement in the quality of life of patients [6], .

Monitoring clinical and diagnostic progression of patients on anti-retroviral treatment (ART) is important to examine responses to the treatment and for clinical decision-making. Patient responses and clinical diagnostic measures to anti-retroviral treatment show differences based on individual and population characteristics, and the type of setting where treatment is delivered [4], [9]. These include drug resistance levels, adherence to treatment, age, source of infection and substance use among others [3], [4], [10], [11], [12]. In Ethiopia, despite an increasing trend of access to ART in the past decade, there is paucity of studies examining the weight and immunological responses of patients in the course of treatment. This study therefore aims to examine predictors of change in CD4 lymphocyte count and weight among HIV infected patients on antiretroviral treatment in country.

## Methodology

### Ethical Consideration

Ethical clearance was obtained from the Institutional Research Ethics Review Committee (IRERC) of Haramaya University. Further approval was obtained from the hospital management before accessing the patients' medical records. In Ethiopia, patients are not customarily requested for consent before their medical record is to be used for research. This includes at the beginning of the recording process and afterward. Initially we wanted to contact loss to follow up patients through nurses to check if they are alive or dead, however the IREC advised us against this citing interference and emotional burden to patients and their families. As a result, we abandoned this approach and collected only non-identifying data from patients' cards. This was done by anti-retroviral treatment nurses working in the respective hospitals in order to assure confidentiality. The IRERC has approved this procedure.

### Study setting

The study was conducted in Hiwot Fana, Jugal and Dil Chora hospitals located in eastern Ethiopia. According to the 2007 ART guideline of Ethiopia, the eligibility criteria for initiation of ART are dependent on the availability of CD4 count instruments in the treating health facilities. When CD4 count is not available, ART can be initiated to patients in WHO clinical stages III and IV irrespective of total lymphocyte count (TLC), and WHO clinical stage II patients if TLC is less than 1,200/ml. Whereas in the presence of CD4 count measurement instruments in the health facilities, treatment is given to WHO clinical stage IV patients, irrespective of the CD4 count – to WHO clinical stage III, if the CD4 cell count is less than 350/mL; and all WHO clinical stages if the CD4 cell count is below 200/mL [13]. The first line treatment regimens recommended in the national guideline are TDF+FTC+EFV or ZDV+3TC+EFV or ZDV+3TC+NVP. There are also alternative treatment regimens like D4T+3TC+NVP, TDF+3TC+NVP, D4T+3TC+EFV, ABC+3TC+NVP, ABC+3TC+EFV and ABC+3TC+ZDV. Usually, once treatment is recommended for AIDS cases by physicians, nurses trained for 1–3 months provide ART treatment, counseling and follow up. Physicians are available for consultation in case of complications and advanced disease patients that need to be admitted to medical wards.

### Study design

A retrospective cohort study was conducted among HIV patients on ART. Data was collected from September to November 2010. A random sample of 1540 patients who started treatment between September 11, 2005 and September 10, 2008 were selected and retrospectively followed until September 10, 2010. The patients' identification numbers were used to extract the necessary data from the database and cards of the HIV patients in the ART clinics.

Socio-demographic characteristics, baseline and follow up clinical and laboratory data, and treatment outcomes were abstracted from patients' cards. The primary outcomes for this study were CD4 lymphocyte count and body weight measurements across time.

### Data collection and quality control

A standard questionnaire was used to extract the data from patients' cards. This form is developed using the standardized ART entry and follow up form employed by the ART clinics. Four advanced ART nurses who were trained on comprehensive HIV care and treatment services and were involved in patient follow ups at the time of the study collected the data. Data collection was supervised by the researchers. All completed data collection forms were examined for clarity and consistency. Death was ascertained by reviewing cards and death certificates. Patients who died from unrelated diseases or accidents, as well as those alive at the end of the follow up period were considered censored. CD4 lymphocyte count (mm^3^) and weight (kg) were measured every six months by the ART nurses, but in some instances such as dysfunction of CD4 counter machines measurements could be extended to longer intervals.

### Data analysis

Descriptive statistics such as median, standard deviations (SDs) and tables were used to investigate the characteristics of the cohort. Descriptive statistics were analyzed using SPSS version 16, while regressions were fitted using STATA version 11. Linear regression was used to characterize and screen data for problems of multicollinearity. Mixed models regression was used to examine changes in CD4 cell count and weight after the baseline measurement. Models were fitted to both the raw and transformed data that approximate to a normal curve. Data transformations ranging from inverse to square roots of the CD4 counts and weight were attempted. Both the random intercept and slope-intercept models were examined to see the model fit. To check the reliability of the model based on the 60^th^ month follow up due to changing missing values, outcomes up to the 48^th^ and 54^th^ months were also fitted and compared with. Model fit was examined using the differences in maximum likelihoods of the competing models. The final model was random slopes and intercepts model in which the slopes as well as the coefficients were allowed to vary. The variables were entered in a simultaneous manner. Due to the large number of models that needed to be checked in addition to comparing random intercept or slope models, we did not use step wise models since they increase the risk of a false positive result (type I error) [14]. Regression coefficients of the final model and their 95% confidence intervals were used as measures of association between the predictors and outcome variable. A p-value of less or equal to 0.05 was considered statistically significant.

## Results

### Characteristics of patients

A total of 1540 patients were included in the study. The median (IQR) age of participants was 32 (28.0–40.0) years and 37.3% (574) were males. Three hundred forty eight (22.7%) and 890 (58.2%) patients were on the WHO clinical stages of II and III at baseline respectively. The median (Inter-quartile range, IQR) of baseline CD4 count was 135 (76.0–198.3) per milliliter (Table 1).

**Table 1:** Baseline characteristics of HIV infected patients initiating antiretroviral therapy.*

	All patients, N¥ (%)	Patients with CD4 count ≤200 (n = 1149)	Patients with CD4 count >200 (n = 361)
Sex			
Male	574 (37.3)	431 (37.6)	131 (36.3)
Female	963 (62.5)	715 (62.4)	230 (63.7)
Age (Median, IQR)	32 (28–40)	32 (28.0–40.0)	35 (28.0–40.0)
Religion			
Muslim	305 (19.8)	220 (19.1)	76 (21.1)
Orthodox	1074 (69.9)	812 (70.7)	241 (66.8)
Protestant	144 (9.4)	103 (9.0)	41 (11.4)
Others	14 (0.9)	14 (1.2)	3 (0.8)
Education			
No education	261 (17.0)	185 (16.1)	67 (18.6)
Primary	681 (44.3)	490 (42.7)	178 (49.3)
Secondary	500 (32.5)	391 (34.1)	102 (28.3)
Tertiary	96 (6.2)	81 (7.1)	14 (3.9)
Marital status			
Never Married	287 (18.6)	217 (18.9)	65 (18.0)
Married	606 (39.4)	475 (41.4)	124 (34.3)
Separated	288 (18.7)	206 (17.9)	74 (20.5)
Divorced	116 (7.5)	89 (7.8)	22 (6.1)
Widowed	242 (15.7)	161 (14.0)	76 (21.1)
Occupation			
Merchant	75 (7.4)	60 (7.7)	15 (6.7)
Gov. Employee	157 (15.5)	122 (15.6)	33 (14.8)
Non-Gov. Employee	41 (4.1)	34 (4.4)	7 (3.1)
Day Laborer	174 (17.2)	130 (16.7)	44 (19.7)
Job-less	402 (39.7)	303 (38.8)	94 (42.2)
Other	163 (16.2)	131 (16.8)	30 (13.5)
Past co-trimoxazole treatment			
Yes	758 (49.2)	576 (50.1)	171 (47.4)
No	782 (50.8)	573 (49.9)	190 (52.6)
WHO stage at baseline			
Stage I	92 (6.0)	76 (6.7)	15 (4.2)
Stage II	348 (22.7)	282 (24.7)	63 (17.5)
Stage III	890 (58.2)	633 (55.5)	239 (66.6)
Stage IV	200 (13.1)	150 (13.1)	42 (11.7)
Weight at baseline in Kgs, median (IQR)	50.0 (44.0–56.0)	50 (44.0–56.0)	50 (45.0–57.0)

¥ Number and percentages unless indicated otherwise. IQR, inter-quartile rage.

* The frequency in cells may be lesser than the cohort size (n = 1540) due to missing values on each of the variables reported.

### Follow up outcomes of the cohort

From the registered patients in this cohort analysis, the outcome of patients as active, deceased, lost to follow up and transferred out were 1005 (67.2%), 86 (5.9%), 210 (14.0%) and 192 (12.8%) respectively.

### Changes in CD4 lymphocyte count and weight

**Figure 1:**
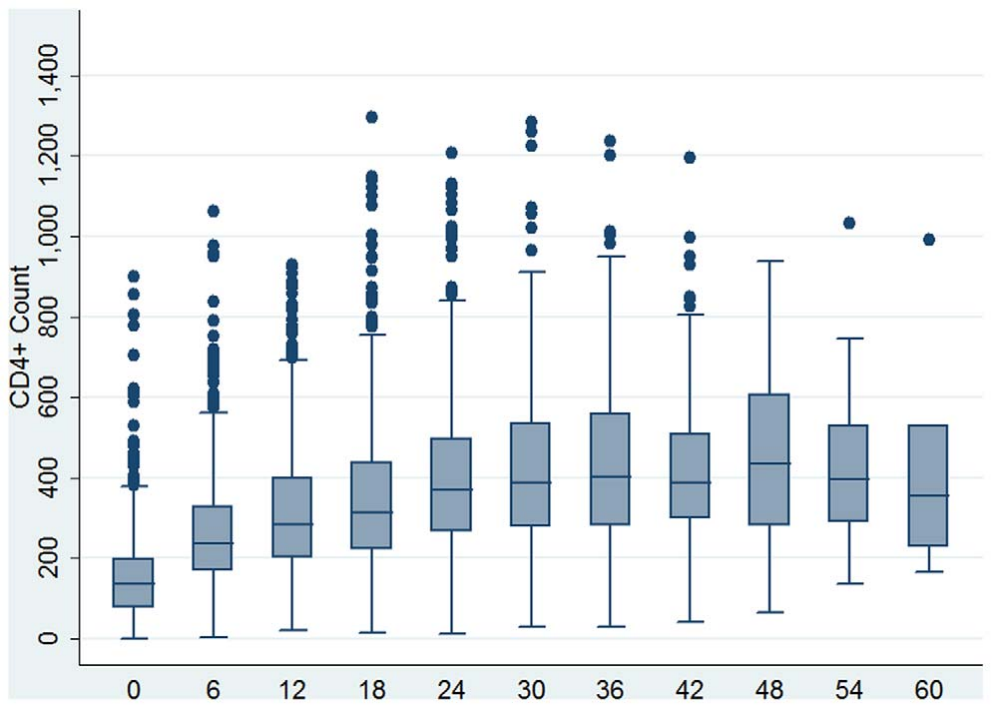
Box plot of changes in CD4+ count among the cohort of patients on anti-retroviral treatment across months of retrospective follow up over five years.

**Figure 2:**
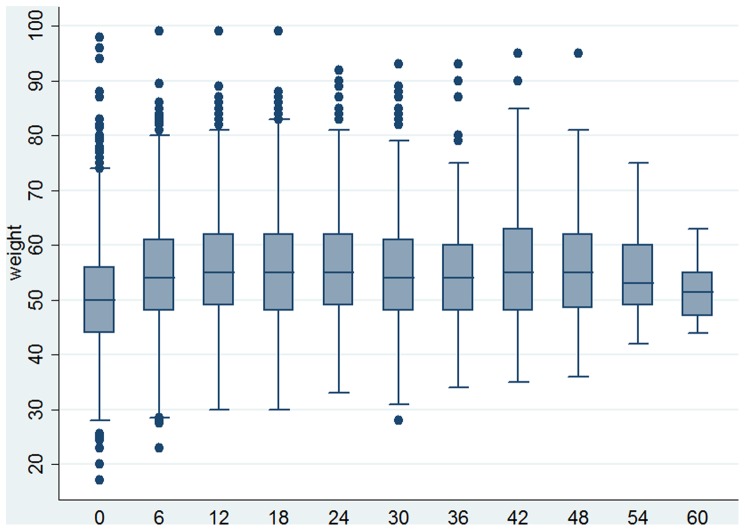
Box plot of changes in weight among the cohort of patients on anti-retroviral treatment in months of retrospective follow up over five years.

As shown in Figure 1, the median CD4 lymphocyte count had improved over the five year period except at the 54^th^ and 60^th^ months where the median CD4 cell count showed a slight decline (Figure 1 and Table 2). In figure 2 we show the changes in body weight over time. The median weight improved after the 6^th^ month follow up period and declined in the 54^th^ and 60^th^ months but they were still higher than that of the baseline values (Figure 1 and Table 2). The random effects models regression was fitted to both the raw and the square root of CD4 lymphocyte count and patients body weight. The length of time in ART (Beta  = 7.91; 95% CI 7.48–8.34; p value 0.000) rather than other variables predicted improvement in CD4 counts. However, actively working patients had better CD4 lymphocyte count than ambulatory patients (P-value 0.038). The model from the square root transformed data was used for interpretation since functional status was significant; the model from the raw data is shown additionally in Table 3 to facilitate comparison. To examine sensitivity of the model to missing values we refitted it using observations up to the 48^th^ and 54^th^ months. However, we did not find differences with the model based on 60^th^ month follow up (Table 3).

**Table 2:** Baseline and follow up values of weight and CD4 count.*

Follow up period	Weight (in kilo grams)		CD4 count	
	N	median (IQR)	N	median (IQR)
Baseline	1489	50.0 (44.0–56.0)	1510	135.0(76.0–198.3)
6^th^ month	1181	54.0 (48.0–61.0)	1098	237.0 (169.0–328.0)
12^th^ month	1088	55.0 (49.0–62.0)	954	285.0 (200.0–398.0)
18^th^ month	971	55.0 (48.0–62.00)	875	315.0 (221.0–480.0)
24^th^ month	784	55.0 (49.0–62.0)	698	370.0 (266.8–498.3)
30^th^ month	562	54.0 (48.0–61.0)	509	387.0 (278.0–536.5)
36^th^ month	351	53.0 (48.0–60.0)	305	403.0 (279.5–559.5)
42^nd^ month	197	55.0 (48.0–63.0)	180	387.5 (298.8–508.5)
48^th^ month	80	55.0 (48.3–62.0)	74	435.0 (279.3–606.0)
54^th^ month	19	53.0 (49.0–60.0)	17	396.0 (287.5–577.5)
60^th^ month	6	51.5 (46.3–57.0)	6	355.0 (213.0–644.8)

* The frequency in cells may be lesser than the cohort size (n = 1540) due to missing values on each of the variables reported.

**Table 3:** Baseline variables associated with changes in CD4 count among the cohort.*

Model 1	CD4 count (non-transformed)			
Predictor variables	Coefficient	SE	95% CI	P-value
Age	−0.97	0.59	−2.13, 0.18	0.099
Hgb level	0.003	0.66	−1.28, 1.29	0.994
WHO stage				
Stage I	ref	ref	Ref	Ref
Stage II	9.28	23.31	−36.40, 54.97	0.690
Stage III	25.63	22.73	−18.91, 70.18	0.259
Stage IV	15.69	28.55	−40.26, 71.64	0.583
Weight	0.12	0.51	−0.88, 1.12	0.818
Functional status				
Working	ref	ref	Ref	Ref
Ambulatory	−20.70	12.98	−46.15, 4.75	0.111
Bedridden	−43.28	29.73	−101.56, 14.99	0.146
Time of visit in months	7.91	0.22	7.48, 8.34	0.000
Model 2	Based on square root transformation of CD4 count measurements			
Age	−0.018	0.02	−0.05, 0.02	0.295
Hgb level	0.001	0.02	−0.04, 0.04	0.975
WHO stage				
Stage I	ref	ref	Ref	Ref
Stage II	0.18	0.68	−1.15, 1.51	0.789
Stage III	0.65	0.66	−0.64, 1.95	0.323
Stage IV	0.006	0.83	−1.62, 1.63	0.994
Weight	−0.002	0.01	−0.03, 0.03	0.886
Functional status				
Working	ref	ref	Ref	Ref
Ambulatory	−0.78	0.38	−1.52, −0.04	0.038
Bedridden	−1.62	0.86	−3.31, 0.06	0.058
Time of visit in months	0.24	0.01	0.23, 0.25	0.000

* Model 2 was used for interpreting the results; the findings are based on all observations of CD4 counts up to the 60^th^ month.

The length of stay on ART was an important predictor of improvement in weight (Beta 0.15; 95% CI 0.13–0.18; p-value 0.000). Advanced WHO clinical stages, lower baseline CD4 lymphocyte count, and baseline hemoglobin levels were associated with lower body weight. Higher age predicted improvement in weight (Beta 0.15; 95% CI 0.07–0.23; p-value 0.000). The model from the square root transformed data revealed similar findings with the one based on the raw data. We show both models in the tables. To examine sensitivity of the model to missing values we refitted it using values of observations up to the 48^th^ and 54^th^. Since these models were not different from the model based on the 60^th^ month observations, the final model included all observations up to the 60^th^ month of follow up (Table 4).

**Table 4:** Baseline variables associated with changes in weight (in kilograms) among the cohort.*

Model 1	Based on non-transformed weight measurements			
Predictor variables	Coefficient	SE	95% CI	P-value
Age	0.15	0.04	0.07, 0.23	0.000
Hgb level	0.11	0.04	0.02, 0.19	0.013
WHO stage				
Stage I	ref	ref	Ref	ref
Stage II	−4.69	1.59	−7.82, −1.57	0.003
Stage III	−3.80	1.56	−6.85, −0.74	0.015
Stage IV	−3.94	1.93	−7.72, 0.15	0.042
CD4 count	0.01	0.00	0.01, −0.00	0.021
Functional status				
Working	ref	ref	Ref	ref
Ambulatory	−4.50	0.88	−6.23, −2.77	0.000
Bedridden	−7.66	1.83	−11.25, −4.06	0.000
Time of visit in months	0.15	0.01	0.13, 0.18	0.000
Model 2	Based on square root transformation of weight measurements			
Age	0.01	0.00	0.00, 0.01	0.000
Hgb level	0.01	0.00	0.01, 0.01	0.016
WHO stage				
Stage I	ref	ref	Ref	ref
Stage II	−0.29	0.11	−0.51, −0.08	0.006
Stage III	−0.24	0.11	−0.45, −0.03	0.006
Stage IV	−0.27	0.13	−0.53, −0.01	0.040
CD4 count	0.0004	0.00	0.00, 0.00	0.012
Functional status				
Working	ref	ref	Ref	ref
Ambulatory	−0.31	0.06	−0.42, 0.19	0.000
Bedridden	−0.53	0.13	−0.78, −0.28	0.000
Time of visit in months	0.01	0.00	0.01, 0.01	0.000

* Model 1 was used for interpretation; the findings are based on all observations of CD4 counts up to the 60^th^ month.

## Discussion

This longitudinal study aimed to examine the progress of CD4 lymphocyte count and weight, and their predictors among a cohort of patients taking antiretroviral treatment in eastern Ethiopia. Our findings indicate that the duration of ART was an important predictor of improvements in CD4 lymphocyte count (beta 7.91; 95% CI 7.48–8.34; p 0.000) and weight (beta 0.15; 95% CI 0.13–0.18; p 0.000). Patient's functional status at the beginning of treatment also predicted improvements in both body weight and CD4 lymphocyte count. Furthermore, higher age, earlier WHO clinical stage, higher CD4 count and high hemoglobin levels were associated with improvements in weight.

The research literature indicates that declines in CD4 lymphocyte count and weight loss among patients on anti-retroviral treatment are associated with increased mortality and morbidity manifesting in the form of deterioration of clinical conditions and decreased functional status in both adults [15] and children [16]. Similarly, in a previous study by our research group, the increment of CD4 count and weight gain were found to be important factors predicting patient survival [17]. Silva and colleagues reported that protease-inhibitor-containing antiretroviral therapy is associated with a weight gain and improvements in body mass index (BMI) [18]. In a cohort study, Mangili and colleagues showed that lower CD4 lymphocyte cell counts were associated with lower weight. When Mangili *et al.* classified patients being provided nutritional supplementation into CD4+ cell count strata, each 100-cell/mm3 decrease in CD4+ cell count was associated with an average of 1.9 kg reduction in weight [19].

Studies have shown that longer duration on ART leads to an improvement of CD4 lymphocyte counts. Similarly, in this study, longer duration on treatment was significantly associated with improvements in CD4 count and weight. A study among 358 patients on ART from southern Ethiopia has documented similar findings [20]. In a multi-country study among sub-Saharan African patients, median CD4 cell counts increased from a baseline of 97 cells/μl to 261 cells/μl at 48 weeks; whereas the proportion of patients with a CD4 cell count <100 cells/μl was reduced from 50% at baseline to 4% at 48 weeks [21]. The main reason for clinical improvement associated with longer duration on therapy is thought to be due to the reduction in viremia [22]. Patients that are able to stay longer on treatment are also more likely to have other parallel healthy behaviors like adherence to treatment as well as social support that improve their clinical outcomes.

In this study we found that older age is associated with improvement in weight. A similar finding has been reported by Mariz and colleagues where older age among patients on ART was associated not only with increased weight gain but also with becoming overweight among patients in Brazil [23]. They reported that patients more than 40 years of age were at risk of overweight or even obesity. Among patients on ART in Tanzania, Li and colleagues found a slightly higher odds of weight loss among patients between the ages of 15 and 29, and patients above the age of 50 three months after initiation of treatment [24]. However, it is difficult to interpret the latter study since its report is based on short term measurements (3 months), while in this study we reported outcomes that are based on follow up of patients for up to 5 years. This highlights a need for future research to examine the relationship between age and weight among patients on ART.

We found that high baseline hemoglobin level predicts improvement in weight gain. Our finding is similar to previous literature in that BMI levels are predicted by not only CD4 counts but also by anemia and hemoglobin levels [23]. For instance, a study carried out among Rwandan women shows risk factors for anemia to be associated with lower body mass index (OR 3.4; 95% CI 2.4–4.1), and CD4 lymphocyte counts below 200 cells/μL (OR 2.41; 95% CI 2.01–3.07). While patients were on treatment, the mean hemoglobin level of 10.9 at ART initiation significantly increased to 12.3 in eight months' time (P<0.001) [25]. However, reverse causation was also documented in another study; lower hemoglobin level at baseline was associated with the risk of significant weight loss at 3 months after initiation among patients on ART in Tanzania [26]. However, it seems that, without regard to the causal relationship between weight gain and anemia, a targeted nutritional supplementation and counseling could help in both weight gain and reduction of anemia among patients.

This study has some limitations. CD4 lymphocyte counts were not available for all patients due to lack of resources at the hospitals where the study was conducted. On top of this HIV RNA measurements were not collected since they are not part of the clinical monitoring of patients in Ethiopia because of their very prohibitive cost. Similarly, diagnostic tests that would confirm the presence of certain opportunistic infections were also limited. Due to this we were not able to include them in our analysis to examine and account for their potential contribution to CD4 count and weight changes. The retrospective study design limited our ability to gather data about factors that may influence morbidity, CD4 count and weight levels, such as food insecurity, social support and depression. However, our study has strengths in that we used a relatively large sample size, multiple referral hospitals, a robust analysis and a longer follow up period. It also has strengths in that it provides evidence about the implementation of ART and its impacts on clinical indicators in the context of treatment provided by lower level health workers in a resource poor country.

We detected a substantial increment in weight and CD4 lymphocyte count among the patients who were taking ART in eastern Ethiopia. The findings indicate that the length of time patients stay on ART and patients' functional status improves both CD4 count and weight gain. Higher age groups, earlier WHO stage, higher CD4 count and high hemoglobin levels were associated with improvements in weight. Ambulatory patients during the initiation of ART should be monitored carefully for immunological response. Patients who are in old age, with low CD4 lymphocyte count, late stage of WHO and lower hemoglobin level may need special diet considerations such as food by prescription programs to help them gain weight over time.

## References

[1] UNAIDS (2010) Global report: UNAIDS report on the global AIDS epidemic 2010. Geneva: Joint United Nations Programme on HIV/AIDS (UNAIDS).

[2] Levy JA (1993) HIV pathogenesis and long-term survival. AIDS 7: 1401–1410. 8280406 10.1097/00002030-199311000-00001

[3] Jacobson LP, Phair JP, Yamashita TE (2004) Virologic and immunologic response to highly active antiretroviral therapy. Curr HIV/AIDS Rep 1: 74–81. 16091226 10.1007/s11904-004-0011-1

[4] Yamashita TE, Phair JP, Muñoz A, Margolick JB, Detels R, et al (2001) Immunologic and virologic response to highly active antiretroviral therapy in the Multicenter AIDS Cohort Study. AIDS 15: 735–746. 11371688 10.1097/00002030-200104130-00009

[5] Resino S, Bellón JM, Sánchez-Ramón S, Gurbindo D, Ruiz-Contreras J, et al (2002) Impact of antiretroviral protocols on dynamics of AIDS progression markers. Arch Dis Child 86: 119–124. 11827906 10.1136/adc.86.2.119 PMC1761061

[6] Siegel K, Lekas H-M (2002) AIDS as a chronic illness: Psychosocial implications. AIDS 16: S69–S76. 12699002 10.1097/00002030-200216004-00010

[7] Palella FJ Jr, Delaney KM, Moorman AC, Loveless MO, Fuhrer J, et al (1998) Declining morbidity and mortality among patients with advanced human immunodeficiency virus infection. HIV Outpatient Study Investigators. N Engl J Med 338: 853–860. 9516219 10.1056/NEJM199803263381301

[8] Murphy EL, Collier AC, Kalish LA, Assmann SF, Para MF, et al (2001) Highly active antiretroviral therapy decreases mortality and morbidity in patients with advanced HIV disease. Annals of Internal Medicine 135: 7–26. 10.7326/0003-4819-135-1-200107030-00005 11434728

[9] Oyomopito R, Lee MP, Phanuphak P, Lim PL, Ditangco R, et al (2010) Measures of site resourcing predict virologic suppression, immunologic response and HIV disease progression following highly active antiretroviral therapy (HAART) in the TREAT Asia HIV Observational Database (TAHOD). HIV Medicine 11: 519–529. 20345881 10.1111/j.1468-1293.2010.00822.x PMC2914850

[10] Hirschel B, Opravil M (1999) The year in review: antiretroviral treatment. AIDS 13: S177–S187. 10885775

[11] Huruy K, Kassu A, Mulu A, Wondie Y (2020) Immune restoration disease and changes in CD4+ T-cell count in HIV-infected patients during highly active antiretroviral therapy at Zewditu memorial hospital, Addis Ababa, Ethiopia. AIDS Research and Therapy 7. 10.1186/1742-6405-7-46 PMC3022664 21176160

[12] Modjarrad K, Vermund SH (2010) Effect of treating co-infections on HIV-1 viral load: a systematic review. Lancet Infectious Diseases 10: 455–463. 20610327 10.1016/S1473-3099(10)70093-1 PMC3071714

[13] HAPCO MOH (2007) Guideline for the management of opportunistic infections and anti-retroviral treatment in adolescents and adults in Ethiopia. In: Ethiopian HIV/AIDS Prevention and Control Office, editor. Addis Ababa: Ethiopia Ministry of Health.

[14] Rabe-Hasketh S, Everitt BS (2007) Multiple regression. A handbook of statistical analyses using Stata. Florida: Chapman & Hall/CRC.

[15] Grinspoon S, Mulligan K (2003) Weight loss and wasting in patients infected with human immunodeficiency virus. Clin Infect Dis 36: S69–78. 12652374 10.1086/367561

[16] Verweel G, van Rossum AMC, Hartwig NG, Wolfs TFW, Scherpbier HJ, et al (2002) Treatment with highly active antiretroviral therapy in Human Immunodeficiency Virus Type 1-infected children is associated with a sustained effect on growth. Pediatrics 109: e25. 11826235 10.1542/peds.109.2.e25

[17] Biadgilign S, Reda AA, Digafe T (2012) Predictors of mortality among HIV infected patients taking antiretroviral treatment in Ethiopia: a retrospective cohort study. AIDS Res Ther 9: 15. 22606951 10.1186/1742-6405-9-15 PMC3403909

[18] Silva M, Skolnik PR, Gorbach SL, Spiegelman D, Wilson IB, et al.. (1998) The effect of protease inhibitors on weight and body composition in HIV- infected patients. AIDS 12: 1645– 1651. 10.1097/00002030-199813000-00012 9764784

[19] Mangili A, Murman DH, Zampini AM, Wanke CA (2006) Nutrition and HIV Infection: Review of Weight Loss and Wasting in the Era of Highly Active Antiretroviral Therapy from the Nutrition for Healthy Living Cohort. Clinical Infectious Diseases 42: 836–842. 16477562 10.1086/500398

[20] Tafese Z, Berhan Y, Abebe H (2012) Changes in nutritional, functional and immunological status of HIV-infected adults with antiretroviral therapy. Ethiop Med J 50: 75–87. 22519164

[21] Lawn SD, Myer L, Bekker L-G, Wood R (2006) CD4 cell count recovery among HIV-infected patients with very advanced immunodeficiency commencing antiretroviral treatment in sub-Saharan Africa. BMC Infect Dis 6. 10.1186/1471-2334-6-59 PMC1435908 16551345

[22] Al-Harthi L, Voris J, Patterson BK, Becker S, Eron J, et al (2004) Evaluation of the impact of highly active antiretroviral therapy on immune recovery in antiretroviral naive patients. HIV Medicine 5: 55–65. 14731171 10.1111/j.1468-1293.2004.00186.x

[23] Mariz Cde A, Albuquerque Mde F, Ximenes RA, Melo HR, Bandeira F, et al (2011) Body mass index in individuals with HIV infection and factors associated with thinness and overweight/obesity. Cad Saude Publica 27: 1997–2008. 22031204 10.1590/s0102-311x2011001000013

[24] Li N, Spiegelman D, Drain P, Mwiru RS, Mugusi F, et al (2012) Predictors of weight loss after HAART initiation among HIV-infected adults in Tanzania. AIDS 26: 577–585. 22156968 10.1097/QAD.0b013e32834f9851

[25] Masaisa F, Gahutu JB, Mukiibi J, Delanghe J, Philippé J (2011) Anemia in Human Immunodeficiency Virus–Infected and Uninfected Women in Rwanda. Am J Trop Med Hyg 84: 456–460. 21363986 10.4269/ajtmh.2011.10-0519 PMC3042824

[26] Li N, Spiegelman D, Drain P, Mwiru RS, Mugusi F, et al (2012) Predictors of Weight Loss after Highly-Active Antiretroviral Therapy (HAART) Initiation among HIV-Infected Adults in Tanzania. AIDS 26: 577–585. 22156968 10.1097/QAD.0b013e32834f9851

